# Periodical propagation of torsion in polymer gels

**DOI:** 10.1038/s41598-022-21198-0

**Published:** 2022-10-06

**Authors:** Yuhei Yamada, Yuji Otsuka, Zebing Mao, Shingo Maeda

**Affiliations:** 1 Department of Mechanical Engineering, Tokyo Institute of Technology, 2-12-1 Ookayama Meguro-ku, Tokyo, 152-8550 Japan; 2 Department of Engineering Science and Mechanics, Shibaura Institute of Technology, 3-7-5 Toyosu, Koto-ku, Tokyo, 135-8548 Japan

**Keywords:** Mechanical engineering, Soft materials, Applied physics, Statistical physics, thermodynamics and nonlinear dynamics

## Abstract

Gel actuators have potential in soft robotics. Although gel actuators can realize various motions like contraction, expansion, and bending, most require external inputs such as batteries and circuits. Herein we propose a periodical torsional motion hydrogel driven by chemical energy from the Belousov-Zhabotinsky (BZ) reaction. Our BZ gel system exhibits autonomous motion without a battery. The elastic moduli of the redox states of the BZ gel are investigated using stress–strain analysis. An experimental system, which integrates the BZ gel and two PDMS (dimethylpolysiloxane) rotators, is designed to evaluate torsion angles. The experimental pre-twist angle dependence of the rotary motion is compared with a theoretical rotation model. The results agree qualitatively. This study should contribute to the development of soft actuators without external components.

## Introduction

Polymers and gels that change shape upon electrical stimulation are promising in diverse applications. For example, they can be easily integrated with mechatronic technologies. A current limitation is that stimulus-responsive polymers and gels require an external controller and power source to drive the mechanical motion. In soft robotics, mechanisms, which integrate actuators and batteries, have recently been proposed by actively utilizing chemical reactions^1,2^. In contrast, living organisms possess autonomous functions free from external stimuli, such as a beating heart. If hydrogels could be chemically engineered to display such autonomous functions using only chemical energy without external electronics, soft robots that behave as if they were alive could be realized.

Many studies have investigated the stimulus-responsive properties of hydrogels^3^. These include evaluating their responsiveness to light^4^, electricity^5^, hydraulics^6^, temperature^7,8^, pH^9^, and ions^10^. Stimuli-responsive polymers and gels have been applied to biomimetic actuators and artificial muscles. Our research strives to develop active macroscale gels, which periodically swell and contract due to the Belousov-Zhabotinsky (BZ) reaction (BZ gels)^11–18^.

The BZ reaction can be observed when an organic acid, an oxidizing agent, and a metal catalyst are mixed under acidic conditions. Similar to the Krebs cycle in living organisms, a network of chemical reactions spontaneously forms during the BZ reaction process. Under uniform agitation conditions, the metal catalyst exhibits redox oscillations. Propagation of the oxidation state through the medium at a constant speed is called a "chemical wave." Chemical waves are generated when a BZ reaction solution stands without stirring. Theoretical studies have investigated the rich behaviors of BZ gels^19–21^. These works explained the experimental results using models based on the Oregonator model^22^, which is a classical model of the BZ reaction in a solution system.

Previously, incorporation of the BZ reaction into gels has been investigated to obtain an autonomous oscillation^23^. We developed non-electronic self-walking gels^16^, peristaltic motion^17^, and gel-pumps^11,13^ by controlling their internal structure. We prepared BZ gels by covalently bonding [Ru(bpy)_2_(4-vinyl-4’-methylbpy)]^2+^([Ru]^2+^, bpy = 2,2’-bipyridine), which is the catalyst for the BZ reaction, to the polymer chain, poly-N-isopropylacrylamide (PNIPAAm). The BZ reaction is induced inside the BZ gel when it is immersed in a BZ solution containing an organic acid, an oxidizing agent, and a strong acid. The BZ gel becomes slightly hydrophilic and swells when the ruthenium complex ions are in the oxidized state. Chemical waves are generated within the BZ gel once the gel length is sufficiently longer than the wavelength of the chemical wave. We observed peristaltic motion of the locally swollen area propagating as the chemical wave propagates^24^.

Previously, we designed BZ gel actuators that exploit swelling and contraction. However, a mechanism is necessary to expand the displacement and generate large deformations because the displacement due to swelling and contraction is only a few hundred micrometers. Here, we focus on the periodic change in the elastic modulus of the BZ gel to realize a periodic torsional motion and a new actuator design. We anticipate that this scheme will easily realize a large displacement because changing the arm’s length of the attached rotators should control the rotary displacement. We initially investigate the stress–strain characteristics of the BZ gel. The elastic modulus of the oxidized state is smaller than that of the reduced state, enabling the generation of periodic torsion by pre-twisting the BZ gel. Then we develop and analyze a mathematical model of the torsion in the BZ gel. This model provides a simple picture of the rotation mechanism.

## Results

### Stress–strain analysis

Hydrogels contain solvents in a network of polymer chains. In our BZ gel, the solubility of the polymer copolymerized with the metal catalyst (Ru) depends on the redox state. Consequently, the gel exhibits swelling-deswelling oscillations according to the change in the osmotic pressure. Focusing on the elasticity, the elastic modulus of a rubber usually increases as the density of the crosslinking points increases when the density is sufficiently large^25^. The elastic modulus should decrease in the oxidized state because the BZ gel swells and the density of crosslinking points decreases.

**Figure 1:**
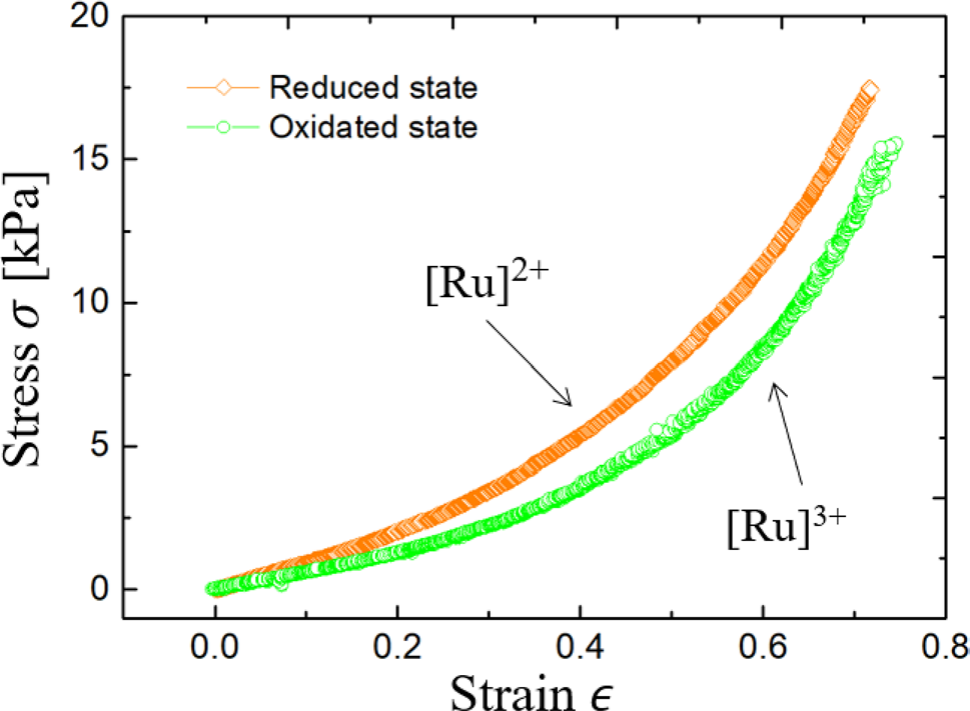
Stress–strain curves of the BZ gel. Orange and green represent the [Ru]^2+^ and [Ru]^3+^ states, respectively.

Figure 1 shows the stretching stress–strain relation of a BZ gel in the fully oxidized state ([Ru]^3+^) and the fully reduced state ([Ru]^2+^). Each state has a different elastic modulus. When the strain is small ($\epsilon < 0.5$), the gel in the oxidized state is more deformable than that in the reduced state. Consequently, we expect that the redox state will realize a difference in the equilibrium angle with a constant torsional stress and subsequently generate a rotary motion.

### Design and model

**Figure 2:**
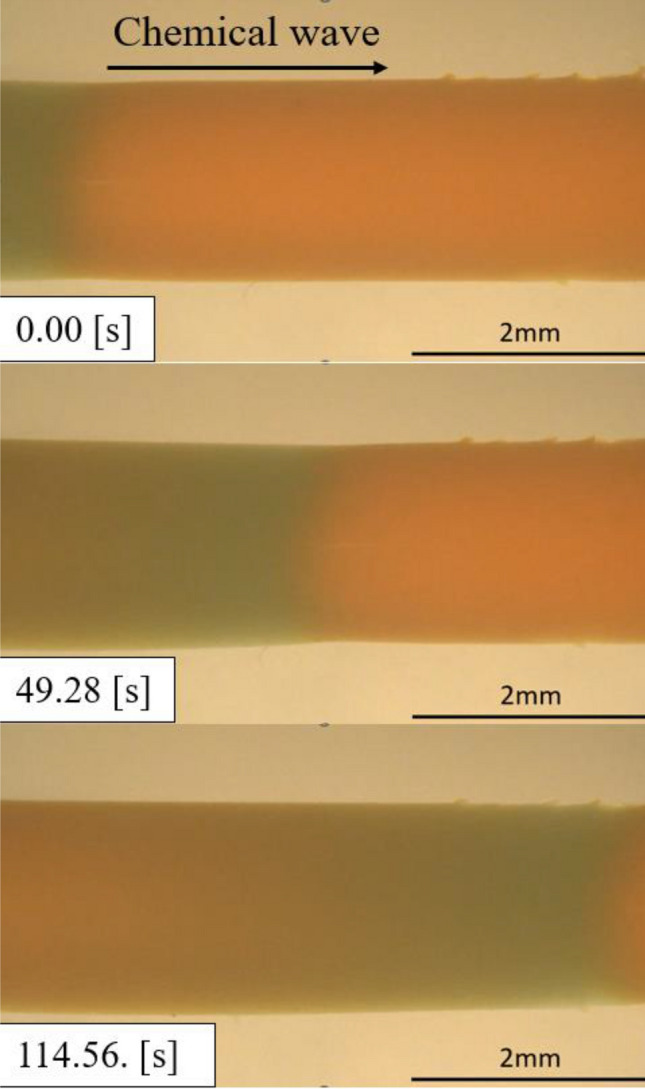
Propagation of chemical waves typically observed in a cylinder-shaped BZ gel. Time is indicated by the number on the left. In this case, the degree of swelling ([diameter in swelling state]/[diameter in contracting state]) $\approx 1.05$, the oscillation period $\approx 301$ s, and the wavelength $\approx 12$ mm.

The BZ gel exhibits various spatial–temporal patterns, which depend on the boundary shape. When the gel is molded as a long cylinder, a chemical wave emerges serially from the end of the gel with a certain period, and the wave propagates to the other end (Fig. 2).

**Figure 3:**
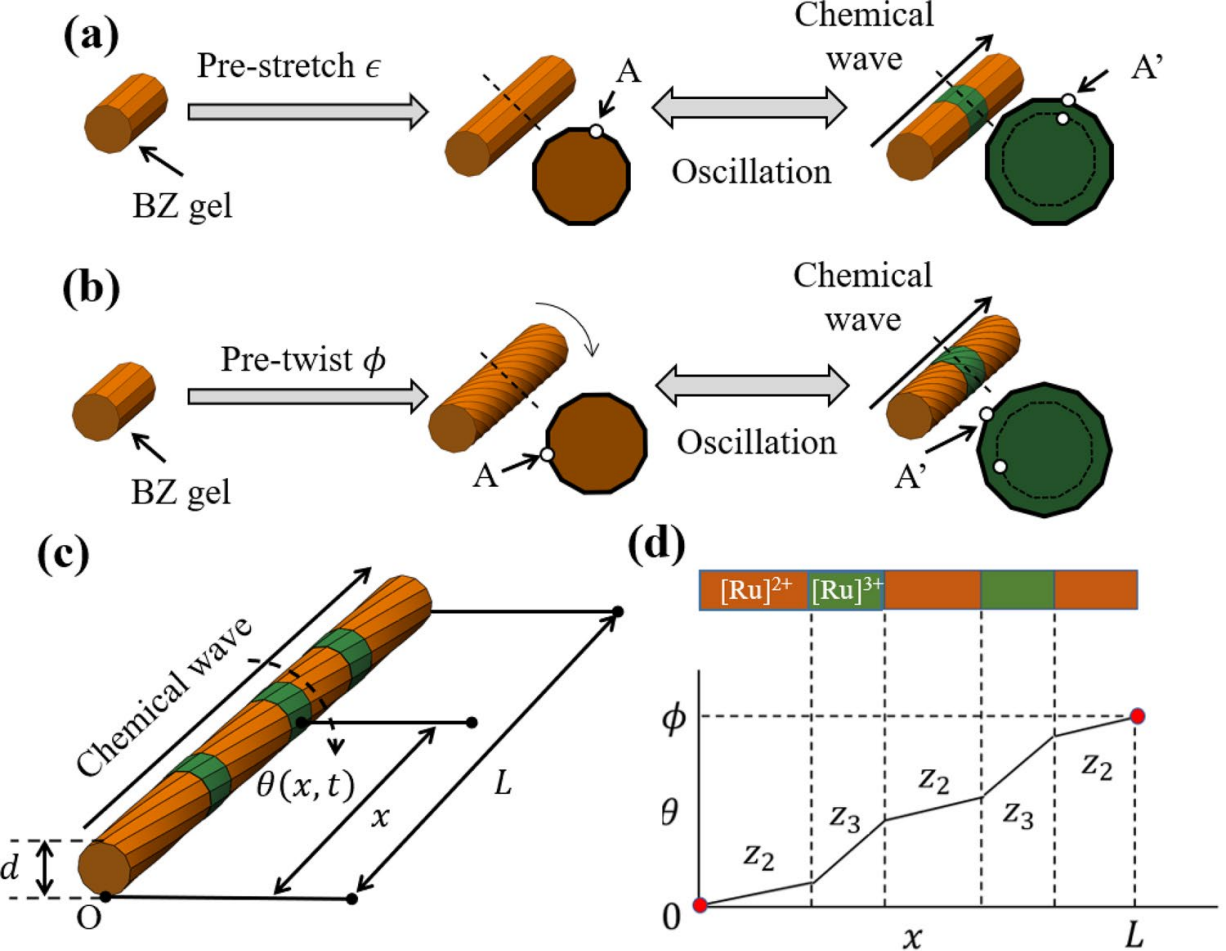
Schematics of the torsional motion of the BZ cylindrical gel with two fixed ends. (**a**) Pre-stretched BZ gel. (**b**) Pre-twisted BZ gel. (**c**) Notations of the pre-twisted BZ gel with wave propagation. (**d**) Schematics of the one-dimensional (1D) twist gel model. The graph depicts $\theta \left( x \right)$ at a certain moment.

Figure 3a and b depict the difference between the pre-deformation schemes for the BZ gel actuators, where $A$ and $A^{\prime}$ indicate the reduced and oxidized states at the same position of the gel, respectively. Previous studies pre-stretched the BZ gels to maximize displacement obtained by the oscillation^11–19^. In these cases, the change in the redox states ($A \leftrightarrow A^{\prime}$) only produces a vertical displacement in the direction of the chemical wave (Fig. 3a). In this study, the BZ gels are pre-twisted, which causes a change in the redox states ($A \leftrightarrow A^{\prime}$) to produce rotational displacement (Fig. 3b).

A simple one-dimensional (1D) model can explain the mechanism. Consider a cylinder-shaped BZ gel with length $L$ and diameter $d$ (Fig. 3c). Assume that the cylinder is sufficiently thin compared with the typical length of the BZ pattern and its axial position specifies the state of the gel piece. $x$ denotes the axial distance from one end of the gel, while $\theta \left( {x,t} \right)$ denotes the angle variation from the non-twisted state at position $x$ and time $t$. $\phi$ denotes the initial pre-twist angle. Since the two ends of the gel are fixed, $\theta \left( {0,t} \right) = 0$ and $\theta \left( {L,t} \right) = \phi$. In the linear regime of torsion of a cylinder, the relation between the applied torque $T$ and the twist angle $\theta$ is given as

1 $$T = \kappa \theta ,$$

where $\kappa$ is the torsion rigidity per length. In this case, the elastic energy $U$ stored by the torsion is expressed as

2 $$U = \frac{\kappa }{2}\theta^{2} .$$

In addition, we assume that (a) the state of the BZ gel (reduced/oxidized) switches the torsion rigidity per length and (b) the torsion angle per length is the same for a piece of gel in the same chemical state. Thus, the rigidity per length and the torsion angle per length of the reduced state ([Ru]^2+^) are denoted as ${\kappa }_{2}$ and ${z}_{2}$, while those of the oxidized state ([Ru]^3+^) are represented as ${\kappa }_{3}$ and ${z}_{3}$, respectively. Furthermore, we assume the mechanical equilibrium state of the torsion in the BZ gel minimizes the elastic energy and the system always maintains mechanical equilibrium. When $a$ and $b$ denote the total length of the reduced state section and the oxidized section, respectively, the total elastic energy is expressed as

3 $$U = \frac{{\kappa_{2} }}{2}\left( {az_{2} } \right)^{2} + \frac{{\kappa_{3} }}{2}\left( {bz_{3} } \right)^{2} ,$$

and the energy minimization condition is

4 $$\frac{\partial U}{{\partial z_{2} }} = \frac{\partial U}{{\partial z_{3} }} = 0.$$

Here, $a$ and $b$ suffice

5 $$a + b = L.$$

Since the total torsion angle throughout the gel is fixed to the pre-twist angle,

6 $$az_{2} + bz_{3} = \phi .$$

From Eqs. (4) and (6), we obtain

7 $$z_{2} = \frac{{\kappa_{3} }}{{a\left( {\kappa_{2} + \kappa_{3} } \right)}}\phi ,$$

8 $$z_{3} = \frac{{\kappa_{2} }}{{b\left( {\kappa_{2} + \kappa_{3} } \right)}}\phi .$$

Because Eqs. (7) and (8) give the torsion angle per length, we can calculate the torsion angle for known patterns of the reduced and oxidized states (Fig. 3d). According to the pattern change by a chemical wave, the torsion angle at a given position varies with time. For a steady time-evolution pattern, the variation of the torsion angle becomes steady. Since ${z}_{2}$ and ${z}_{3}$ are proportional to $\phi$ as given in Eqs. (7) and (8), the torsion angle variation is also proportional to $\phi$, which is consistent with the experimental results.

In a special case when the whole gel is in a uniform state, $a=L$ ($b=L$), Eq. (5) leads to $b=0$ ($a=0$). In this case, Eqs. (7) and (8) become invalid because Eq. (6) gives ${z}_{2}=\phi /L$ when $a=L$ and ${z}_{3}=\phi /L$ when $b=L$, $\theta \left(x,t\right)=\phi x/L$ in both cases. Consequently, angle variation does not occur because $\theta \left(x,t\right)$ is independent of time. Therefore, the periodic torsional motion can only be generated when two regions with different elastic moduli coexist in a single material.

## Experiment and discussion

**Figure 4:**
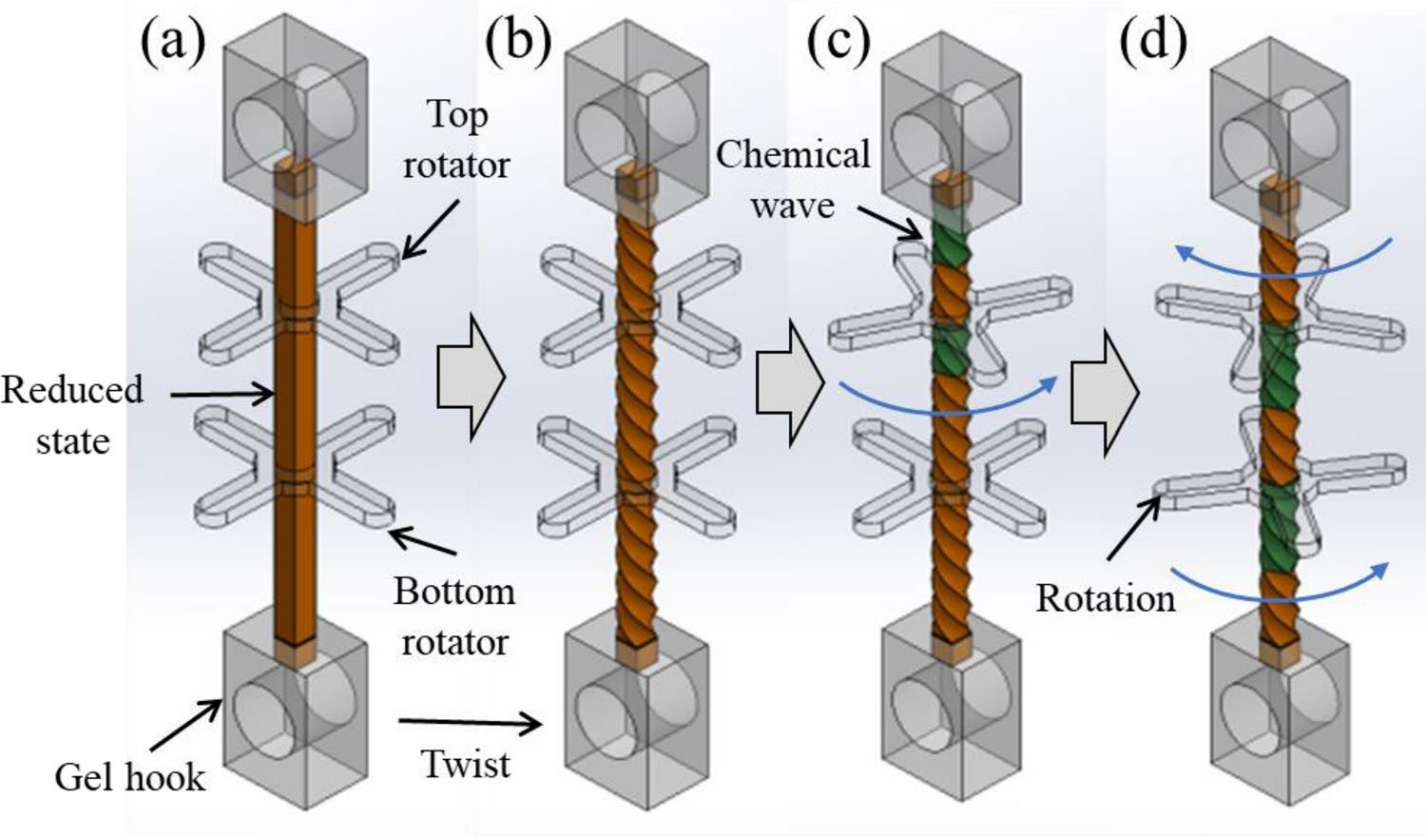
(**a**) Assembly of the BZ gel, top PDMS rotator, bottom rotator, and gel hooks. (**b**) Pre-twist of the BZ gel. (**c**) Counterclockwise rotation of the top rotator as the chemical wave propagates. (**d**) Clockwise rotation of the top rotator and counterclockwise rotation of the bottom rotator. (**a**–**d**) are created by Adobe Illustrator (CC2015) https://www.adobe.com/jp/products/illustrator.html).

We synthesized a cylinder-shaped BZ gel with a diameter $d=1$ mm. Initially, the gel is pre-stretched 150% of the natural length and the two ends were fixed by a gel hook, and two PDMS rotators were attached to the gel (Fig. 4a). The length between the hooks was 10 mm, which corresponds to $L$ in the model, and the length between the rotators was 5 mm. Then the gel was pre-twisted (Fig. 4b). Due to the soft and fragile nature of the gel, the pre-twist was applied slowly. Chemical waves emerge upon immersing the gel in the BZ reaction substrates (Fig. 4c). In accordance with the BZ periodic oscillation, the rotators produce a periodic rotary motion (Fig. 4d). Similar to other BZ reaction systems, the system degrades on a long-time scale. However, degradation was negligible on our experimental time scale (~ 3000 [s]). Although we confirmed the gel can be reused a few times, a newly synthesized gel was used in each experiment.

**Figure 5:**
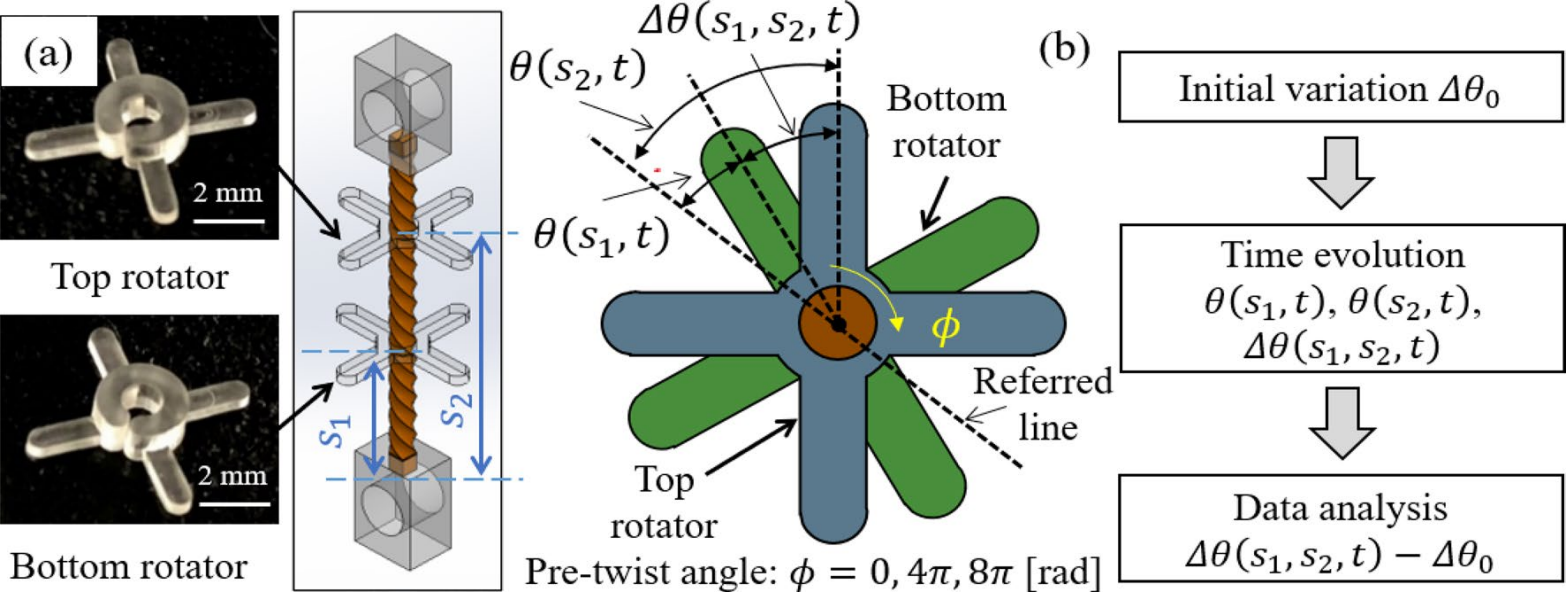
(**a**) Photos of the PDMS rotators and schematic diagram of the calculated angular variation $\Delta \theta \left( {s_{1} ,s_{2} , t} \right)$. (**b**) Data analysis procedure. (Middle image of (**a**) is created by Adobe Illustrator (CC2015) https://www.adobe.com/jp/products/illustrator.html).

To measure the torsion angle driven by the twisted BZ gel, rotators were attached to the gel (Fig. 5). Details are described in the Methods section. We measured the torsion angles of rotators, ${\theta (s}_{1},t)$ and ${\theta (s}_{2},t)$, with respect to the reference line using a Motion Analyzer (Rockwell Automation). Since the initial pattern evolution of the BZ gel is unsteady after immersing in the reaction substrate, we set $t=0$ after the initial behavior stabilized. We estimated the time evolution of the rotational motion by calculating the angular variation $\Delta \theta \left({s}_{1},{s}_{2},t\right)={\theta (s}_{2},t)-{\theta (s}_{1},t)$. Supplementary Video S1 is a demo video of this experiment, which includes the front view and vertical view of the system. Videos S2, S3, S4 are the vertical views of the experiment for $\phi =0, 4\pi , 8\pi$, respectively, and Supplementary Fig. S1 shows an example of the measured angle.

**Figure 6:**
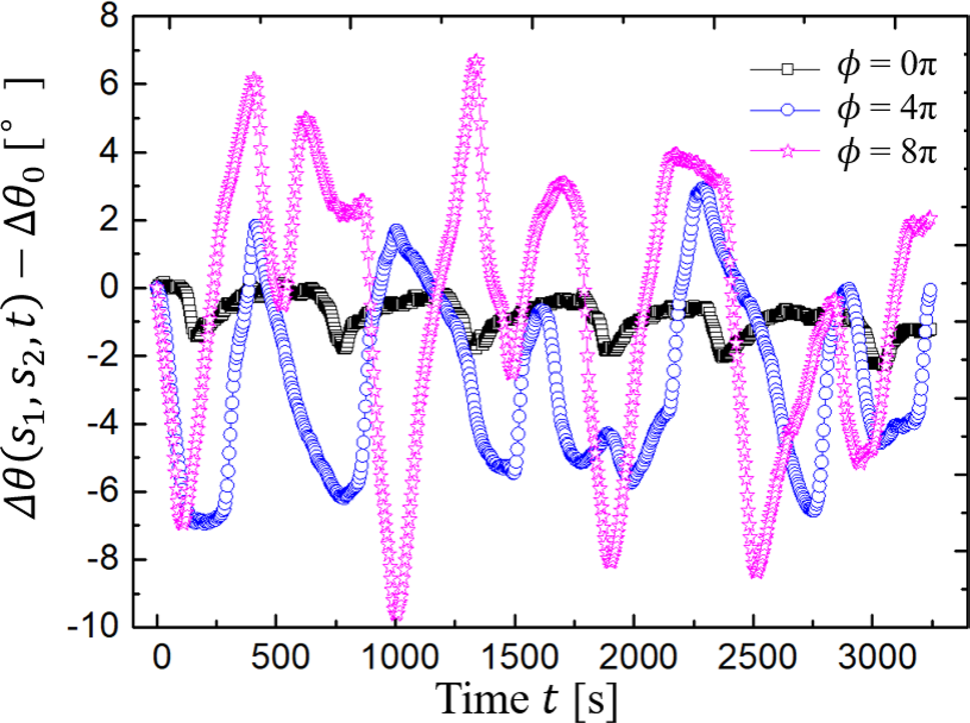
Angular variation $\Delta \theta \left( {s_{1} ,s_{2} ,t} \right) - \Delta \theta_{0}$ of PDMS rotators driven by the BZ gels when the gels are pre-twisted for $\phi = 0\pi , 4\pi ,{\text{and}} 8\pi$ (one realization).

We measured $\Delta \theta \left({s}_{1},{s}_{2},t\right)$ by varying the pre-twist angle $\phi$. Here, the initial angular variation $\Delta {\theta }_{0}\equiv \Delta \theta \left({s}_{1},{s}_{2},0\right)$ depends on the mounting process of the rotators. To eliminate this effect and estimate the net angular variation, we plotted $\Delta \theta \left({s}_{1},{s}_{2},t\right)-\Delta {\theta }_{0}$ to compare the results. Figure 6 shows the time evolution of the angular variation for different pre-twist angles. We conducted experiments for $\phi \le 8\pi$ because our BZ gels break beyond that. As $\phi$ increases, the oscillation behavior of the angular variation becomes irregular. This behavior may be because pre-twisting increases the inhomogeneity of the stress field in the gel. Although the oscillation period varies in each experiment due to the sensitivity to environmental conditions, we confirmed the robustness of the torsional motion. The amplitude of $\Delta \theta \left({s}_{1},{s}_{2},t\right)-\Delta {\theta }_{0}$ increases with increasing $\phi$. Specifically, $\Delta \theta \left({s}_{1},{s}_{2},t\right)$ fluctuates less than 2° without pre-twisting (Fig. 6). When $\phi =4\pi$, $\Delta \theta \left({s}_{1},{s}_{2},t\right)-\Delta {\theta }_{0}$ floats between − 6.9° and 2.9° and when $\phi =8\pi$, it floats between –9.6° and 6.3°.

**Figure 7:**
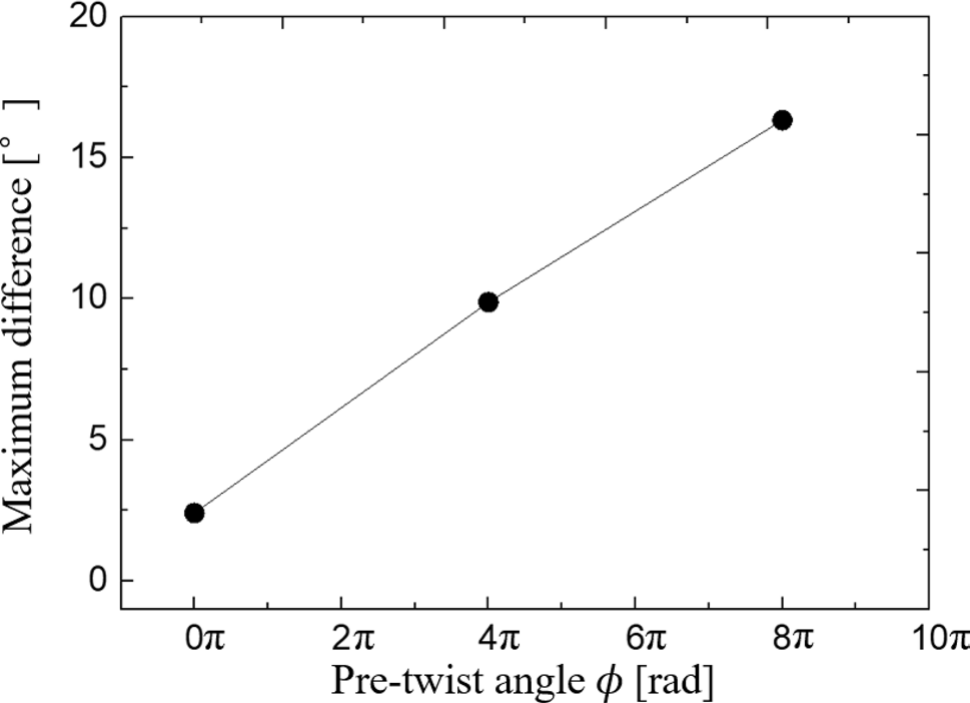
Maximum difference of the angular variation ${\text{Max}}_{t} \left[ {\Delta \theta \left( {s_{1} ,s_{2} ,t} \right)} \right] - {\text{Min}}_{t} \left[ {\Delta \theta \left( {s_{1} ,s_{2} ,t} \right)} \right]$ versus the pre-twist angle $\phi$. Data is the same as that used in Fig. 6.

To quantify the dependency of the angular variation’s amplitude on the pre-twist angle, we calculated ${\mathrm{Max}}_{t}\left[\Delta \theta \left({s}_{1},{s}_{2},t\right)\right]-{\mathrm{Min}}_{t}\left[\Delta \theta \left({s}_{1},{s}_{2},t\right)\right]$, which is the maximum difference of the angular variation over time in the experiment. The maximum difference of angular variation increases proportionally to the pre-twist angle (Fig. 7). The result agrees with our analysis.

## Conclusion

Here, the rotary motion of the pre-twisted BZ gel is elucidated in terms of the wave patterns, the pre-twist angle, and torque generation. First, we designed a system that integrates with the BZ gel and can generate torsional motion. The PDMS rotators were used to evaluate torsion angles. Additionally, we derived a mathematical model for rotary motion generated by the BZ gels assuming the elastic moduli depend on the redox state of the gel. We synthesized BZ gels and their hooks, fabricated two PDMS rotators using a replicating mold, and built a tensile test rig. Finally, we experimentally demonstrated periodic rotary motion. The results qualitatively agree with theoretical predictions.

Controlling the chemical wave profile such as the period and wavelength is crucial for practical applications. However, theoretical simulations suggest that the profile is highly sensitive to the boundary condition^20^. Actually, we observed large fluctuation of the wave’s profile even when the experimental condition is the same. Developing a technique to control the chemical wave and revealing the effect of pre-twist on the wave’s pattern is an important future work. We believe that the periodic torsional motion of the BZ gel should realize a new design methodology for soft robots.

## Methods

### Materials

#### BZ gel

The amplitude of the volume oscillation of a microphase separated BZ gel is much larger than that of a conventional BZ gel. Adding AMPS (2-acrylamido-2-methylpropanesulfonic acid) to a poly(NIPAAm-*co-*[Ru]) gel network generated microphase separated BZ gels. Because polymer-rich domains formed in the microphase separated BZ gels, an effluent pathway for solvents was realized. The polymer-rich domains rapidly aggregated or dispersed in the microphase separated BZ gel. To prepare the poly [NIPAAm-*co*-[Ru]-*co*-AMPS] gel for the BZ gel, NIPAAm (1.91 g), AMPS (0.0606 g), [Ru]^2+^(0.2374 g), N, N’-methylenebisacrylamide (MBAA, 0.0606 g), and 2, 2’-azobis(isobutyronitrile) (AIBN as an initiator, 0.0271 g) were mixed in water and methanol. Then solutions of deionized water and methanol were substituted by nitrogen to remove the oxygen. All the prepared chemicals were mixed and stirred for 24 h to obtain the equilibrium state. The compounds were poured into a capillary tube with an inner diameter of 1.0 mm. The capillary was then sealed at both ends with epoxy putty and thermally polymerized at 60 °C for at least 12 h. Then, the gel was extracted from the glass tube, and it was immersed in the mixed solvents of methanol and pure water (methanol: water = 75, 50, 25, and 0%, one day for each group).

The reaction solution for the BZ reaction was made by mixing nitric acid (6.3 w/v%, 89 ml), sodium bromate (15.0 w/v%, 8.4 ml), and malonic acid (31.2 w/v%, 2.08 ml). In our experiment, the temperature of the solution was maintained at 20 °C.

#### Hook

For the tensile tests, we synthesized a hook made of poly (AAm-*co*-AAc) gel to anchor the ends of the BZ gel. The hook is made of MBAA (0.245 g), acrylic acid (AAc, 5.155 g), acrylamide (AAm, 9.57 g), and α-ketoglutaric acid (0.232 g). They were mixed with pure water treated with nitrogen and stirred for one day. (Here, the mixing process was done in a draft chamber because acrylic acid has a strong pungent odor.) By using the photo-polymerization method, the hooks are synthesized at the two ends of the BZ gel (Supplementary Fig. S2). Note that prior to synthesizing the hook, the BZ gel was immersed in a mixed monomer solution for 1 day.

#### Photo-polymerization

To synthesize hooks (Supplementary Fig. S2), we fabricated two types of molds: PDMS mold (SILPOT 184) and black acrylic masks. Each mold was placed on a glass slide (Fig. S2a). A black mask prevents UV light from penetrating the middle part of the BZ gel. After pouring the mixed monomer solution into the mold (Fig. S2b), the mold was covered with the acrylic mask and the glass slide. The device was then exposed to ultraviolet light (HB100A-1, Seritech) for four minutes (Fig. S2c). Then, a BZ gel with hooks made by the poly (AAm-*co*-AAc) gel is obtained (Fig. S2d). Figure S2e and f show images of the molds and the gels, respectively. The fabricated BZ gel was then immersed in nitric acid (1 M) for two days to be the equilibrium state.

#### Rotator

To fabricate PDMS rotators, the replica molding method was used. The mold was made of an acrylic plate manufactured by a cutting machine (MODELA MDX-40A, Roland). The mold was then adhered to Pyrex glass. PDMS was made by mixing the base (SILPOT184 BASE) and the curing agent (SILPOT184 CAT) at a 1:1 ratio (wt/wt). Next, the prepolymer of the PDMS was fully mixed in a rotary mixer (AR-100, THINKY) and degassed in an aspirator (AS-01, AS ONE) for 15 min. The prepolymer PDMS was poured into the mold, covered with another piece of Pyrex glass, and clamped with a clip. The whole device was placed in a 60 °C oven for 24 h (DX 402, Yamato Scientific). After drying, the PDMS rotators were carefully released from the acrylic plates by a metal ruler.

### Elastic property testing

For the motorized stage of the tensile test, we used SGSP26-200 (Sigma Koki), an XY-axis steel stage TSD-1002S (Sigma Koki), two-axis controller (SHOT-702 Sigma Koki), and BIOS Control PC software. LVS-5GA (KYOWA) was used as a micro-load cell. A water bath (NCB-1200 EYELA Tokyo Rika Instruments) was used to keep the temperature of the system. Photos and videos were captured by a CCD camera (TOSHIBA IK-HR2D) and a Video camera (SONY HDR-CX630), respectively.

We adopted a cutting machine (MODELA MDX-40A, Roland) to manufacture the acrylic plate and molds. To realize fully reduced state ([Ru]^2+^), the BZ gel was immersed in the solution composed of nitric acid solution (20 °C) over 1 h. On the other hand, to realize fully oxidized state ([Ru]^3+^), the gel was immersed in the solution composed of nitric acid and bromic acid (20 °C) over 1 h.

### Torsion angle measurement

A Motion Analyzer (Rockwell Automation) is used to measure the torsion angle of the rotators. The Motion Analyzer detected three marked points in a video to measure the angle. The reference points included a point on the fixed substrate in the system, the longitudinal axis of the gel, and the tip of the rotator. In our experiment, the video was acquired from above, as Supplemental Videos S2−S3.

## Supplementary Information

Supplementary Information 1.

Supplementary Video 1.

Supplementary Video 2.

Supplementary Video 3.

Supplementary Video 4.

## Data Availability

The authors declare that the data supporting the findings of this study are available within the paper.
