# Supplementary material for: Periodical propagation of torsion in polymer gels
Source: Sci Rep. 2022 Oct 6;12:16679. doi: 10.1038/s41598-022-21198-0 (PMC9537306; doi:10.1038/s41598-022-21198-0)
Supplement: Supplementary file 1 — Supplementary Information 1. [file 41598_2022_21198_MOESM1_ESM.pdf]

Periodical propagation of torsion in polymer gels

Yuhei Yamada[1], Yuji Otsuka[2], Zebing Mao[1], Shingo Maeda[1*]

[1]Department of Mechanical engineering, Tokyo Institute of Technology, 2-12-1 Ookayama Meguro-ku Tokyo, 152-8550, Japan.

[2]Department of Engineering Science and Mechanics, Shibaura Institute of Technology, 3-7-5 Toyosu, Koto-ku, Tokyo 135-8548, Japan.

*Corresponding author: maeda.s.ao@m.titech.ac.jp

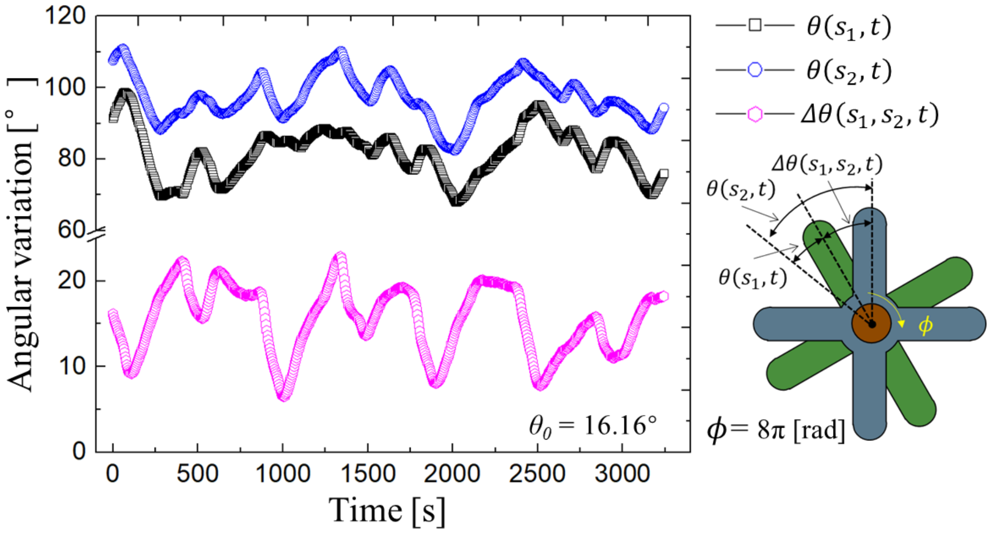

Fig. S1 Angular variation $\theta(s_1,t), \theta(s_2,t), \Delta\theta(s_1,s_2,t)$ of PDMS rotators driven by the BZ reaction when $\phi = 8\pi$ and $\Delta\theta_0 = 16.16°$.

Fig. S1 shows the torsional motion of the BZ gel fluctuates with a pre-twisted angle of 8π. Three angles of $\theta(s_1,t)$, $\theta(s_2,t)$ and $\Delta\theta(s_1,s_2,t)$ fluctuates over the experimental time. The fluctuation of $\theta(s_2,t)$ lags behind $\theta(s_1,t)$ because the BZ wave propagates from the upper rotator to the bottom rotator and meanwhile, the wavelength of BZ gel is smaller than the distance between two rotators. Here, $\Delta\theta(s_1,s_2,t)$ starts with the angle $\Delta\theta_0 = 16.16°$.

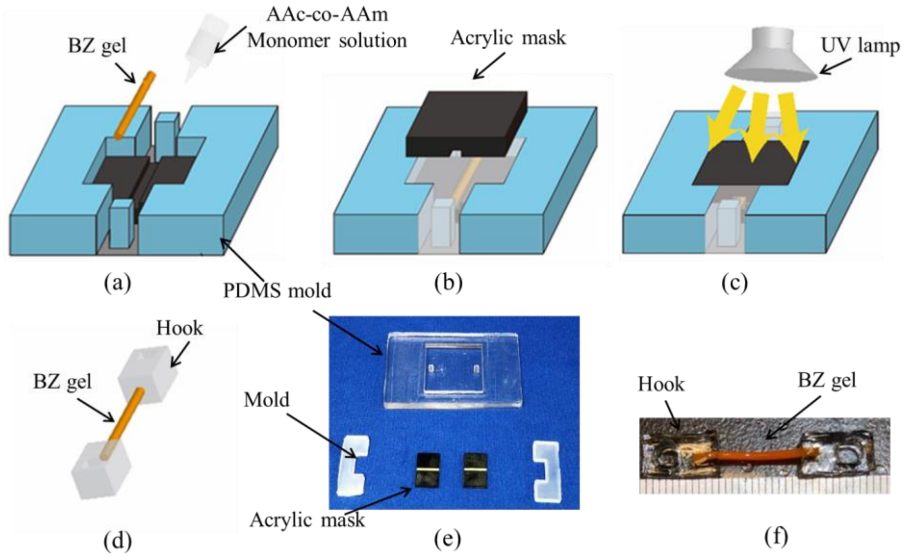

Fig. S2 Synthesis of the BZ gel and AAc-co-AAm gel (hook). (a) Preparation of the BZ gel, Aac-co-AAm (hook), Acrylic mask, and PDMS mold. (b) Placement of upper acrylic mask. (c) UV exposure. (d) Removal. (e) Photo of the prepared molds. (f) Photo of the BZ gel and its hook. ((a)-(d) are created by Adobe Illustrator (CC2015) https://www.adobe.com/jp/products/illustrator.html)

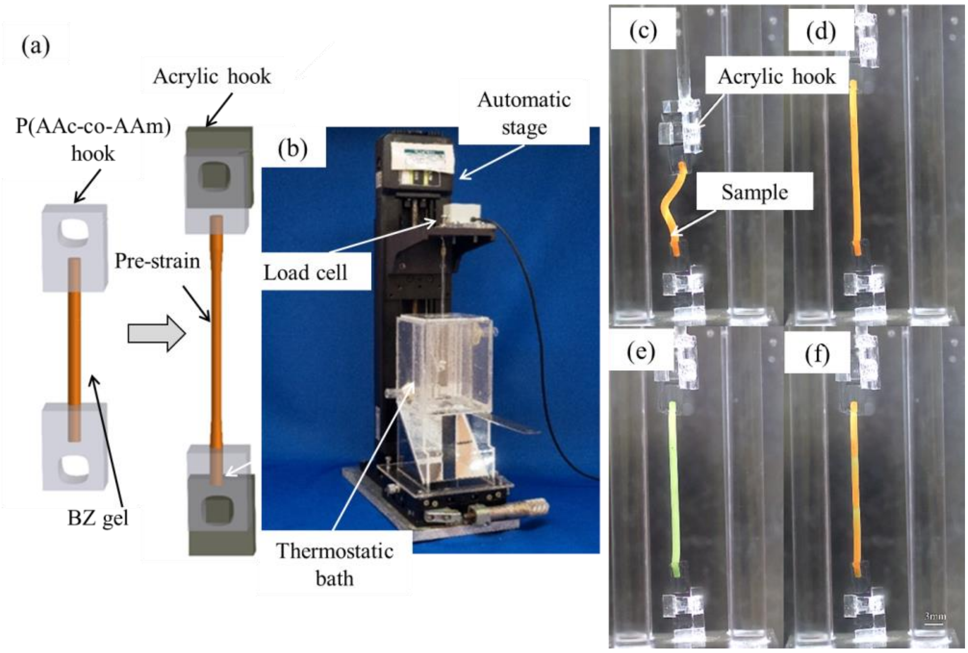

Fig. S3 Experimental setup. (a) Schematic image of the BZ gel with hook. (b) Photo of the tensile test rig. (c) BZ gel hung in the acrylic hook. (d) Pre-stretch process. (e) Addition of BZ reaction solution. (f) BZ waves generation along the axis of the BZ gel. ((a) is created by Adobe Illustrator (CC2015) https://www.adobe.com/jp/products/illustrator.html)
